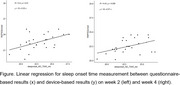# Nocturnal Sleep Measured by Actigraphy and Questionnaires in Care Home Residents Living with Late‐Stage Dementia and Agitation

**DOI:** 10.1002/alz70857_105441

**Published:** 2025-12-25

**Authors:** Ta‐Wei Guu, Dag Aarsland, Dominic H Ffytche

**Affiliations:** ^1^ Division of Psychiatry, Department of Internal Medicine, China Medical University Beigang Hospital, Yunlin, Taiwan; ^2^ Centre for Healthy Brain Ageing, Department of Psychological Medicine, Institute of Psychiatry, Psychology, and Neuroscience, King's College London, London, London, United Kingdom; ^3^ Centre for Age‐Related Medicine (SESAM), Stavanger University Hospital, Stavanger, Norway; ^4^ Institute of Psychiatry, Psychology & Neuroscience, King's College London, London, United Kingdom; ^5^ Centre for Healthy Brain Ageing, Department of Psychological Medicine, Institute of Psychiatry, Psychology, and Neuroscience, King's College London, London, London, United Kingdom, London, London, United Kingdom

## Abstract

**Background:**

Sleep disturbances are common in dementia patients, and nocturnal behavioural and psychological symptoms of dementia (BPSD) are particularly challenging to caregivers. Measuring them currently relies on carer‐rated questionnaires, which can be imprecise. We explore the use of a validated research‐grade actigraphy (Geneactiv Original) and an open‐sourced R‐package GGIR to measure the sleep of care home residents living with late‐stage Alzheimer's disease (AD) and agitation from the Sativex® for the treatment of Agitation & Aggression in Alzheimer's Dementia (STAND) trial (ISRCTN registry: 97163562).

**Method:**

Out of 29 participants from STAND trial, 28 (14 female) accepted wearing the device were included. The participants’ BPSD were assessed with caregiver‐rated Neuropsychiatry Inventory‐Nursing Home version (NPI‐NH) and Pittsburgh Sleep Quality Index (PSQI) at baseline, week 2 and week 4. Sleep‐related variables, including sleep duration, onset and offset time, efficiency, Wake‐After‐Sleep‐Onset (WASO) duration and the Sleep Regularity Index, were calculated using GGIR and then correlated with the NPI and PSQI items of the corresponding period. Spearman correlation and linear regression to analyse questionnaires and device‐based results were undertaken, and a two‐tailed alpha of 0.05 with a *p*‐value < .05 were applied and considered statistically significant.

**Result:**

The prevalence of sleep problem was high, regardless of the measuring tool (62.1% NPI‐sleep item ≥ 4, 72.4% PSQI > 5, and 51.9% actigraphy‐derived sleep efficiency < 85%) and the trial group assigned. Overall, participants' average sleep regularity index was very low (26.4 ± 15.0 during week 1‐2, and 25.7 ± 16.9 during week 3‐4). Questionnaires and actigraphy results averaged across multiple days had good correlations in qualitative variables, but wearables appeared more sensitive in measuring time‐dependent variables and reflecting the sleep regularity. Using a data‐driven approach, a moderate degree of correlation was found between WASO duration and irritability throughout the trial period (*R* = 0.43 and 0.44 over week 1‐2 and week 3‐4, respectively, both *p* <0.05).

**Conclusion:**

Nocturnal sleep appeared commonly poor and irregular in this population, and the irregularity may not be captured by questionnaires. Clinical trials could consider applying wearables as alternative measurements, which might be able to offer information to complement questionnaire‐based results and to explore novel therapeutic targets.